# Sinapic Acid Suppresses SARS CoV-2 Replication by Targeting Its Envelope Protein

**DOI:** 10.3390/antibiotics10040420

**Published:** 2021-04-11

**Authors:** Raha Orfali, Mostafa E. Rateb, Hossam M. Hassan, Mona Alonazi, Mokhtar R. Gomaa, Noura Mahrous, Mohamed GabAllah, Ahmed Kandeil, Shagufta Perveen, Usama Ramadan Abdelmohsen, Ahmed M. Sayed

**Affiliations:** 1 Department of Pharmacognosy, College of Pharmacy, King Saud University, P.O. Box 22452, Riyadh 11495, Saudi Arabia; rorfali@ksu.edu.sa (R.O.); shakhan@ksu.edu.sa (S.P.); 2 School of Computing, Engineering & Physical Sciences, University of the West of Scotland, Paisley PA1 2BE, UK; Mostafa.Rateb@uws.ac.uk; 3 Department of Pharmacognosy, Faculty of Pharmacy, Nahda University, Beni-Suef 62513, Egypt; hossam.mokhtar@nub.edu.eg; 4 Department of Pharmacognosy, Faculty of Pharmacy, Beni-Suef University, Beni-Suef 62513, Egypt; 5 Department of Biochemistry, Faculty of Science, King Saud University. P.O. Box 12372, Riyadh 11495, Saudi Arabia; moalonazi@Ksu.edu.sa; 6 Center of Scientific Excellence for Influenza Virus, Environmental Research Division, National Research Centre, Giza 12622, Egypt; Mokhtar.Rizk@human-link.org (M.R.G.); noura.mahrous1995@gmail.com (N.M.); gaballah09@gmail.com (M.G.); Kandeil_a@hotmail.com (A.K.); 7 Department of Pharmacognosy, Faculty of Pharmacy, Minia University, Minia 61519, Egypt; 8 Department of Pharmacognosy, Faculty of Pharmacy, Deraya University, 7 Universities Zone, New Minia 61111, Egypt

**Keywords:** sinapic acid, SARS CoV-2, COVID-19, viral envelope protein, molecular dynamic simulation

## Abstract

SARS CoV-2 is still considered a global health issue, and its threat keeps growing with the emergence of newly evolved strains. Despite the success in developing some vaccines as a protective measure, finding cost-effective treatments is urgent. Accordingly, we screened a number of phenolic natural compounds for their in vitro anti-SARS CoV-2 activity. We found sinapic acid (SA) selectively inhibited the viral replication in vitro with an half-maximal inhibitory concentration (IC_50_) value of 2.69 µg/mL with significantly low cytotoxicity (CC_50_ = 189.3 µg/mL). Subsequently, we virtually screened all currently available molecular targets using a multistep in silico protocol to find out the most probable molecular target that mediates this compound’s antiviral activity. As a result, the viral envelope protein (E-protein) was suggested as the most possible hit for SA. Further in-depth molecular dynamic simulation-based investigation revealed the essential structural features of SA antiviral activity and its binding mode with E-protein. The structural and experimental results presented in this study strongly recommend SA as a promising structural motif for anti-SARS CoV-2 agent development.

## 1. Introduction

Coronaviruses (family Coronaviridae) are a type of enveloped virus that can infect humans, causing common colds. They are also responsible for various lethal diseases in birds and mammals [1]. Highly infectious members of this family have been previously widespread, producing severe respiratory complications, i.e., SARS and MERS CoVs [2]. At the end of 2019, a new coronavirus (i.e., SARS-CoV-2) strain invaded the globe, causing a severe health disaster. Since the SARS-CoV-2 pandemic declaration, research groups around the world prepared themselves to face the problem. Now, after almost 15 months, they have successfully developed a number of effective vaccines, and trials on antiviral agents are underway [3]. Natural products have shown their potential against several SARS CoV-2 targets [2,4,5,6,7,8]. Besides, most of the essential viral proteins’ structures and functions have been successfully characterized (https://swissmodel.expasy.org/repository/species/2697049, accessed on 23 December 2020) [9]. Hence, utilizing all this structural information in comprehensive structural-based drug discovery and design would eventually lead to finding proper therapeutic agents. 

Ivermectin is an FDA-approved anti-parasitic natural product that wasamong the first reported anti-SARS CoV-2 agents and has also demonstrated antiviral efficacy in the clinical trials [10,11,12]. Later, artemisinin and its congeners also demonstrated a significant in vitro anti-SARS CoV-2 inhibitory activity. Additionally, it was reported to contribute to a faster recovery of COVID-19 patients [13,14]. Both ivermectin and artemisinin were suggested to exert their antiviral activity by blocking the viral entry inside the host cells via several mechanisms [10,11,12,13,14]. Recently, a group of plant-derived phenolic compounds has shown potent in vitro anti-SARS CoV-2 activity via targeting the viral main protease [15,16]. Readers can find more comprehensive information on the previously reported anti-SARS CoV agents in these recent reviews [2,17,18].

**Figure 1:**
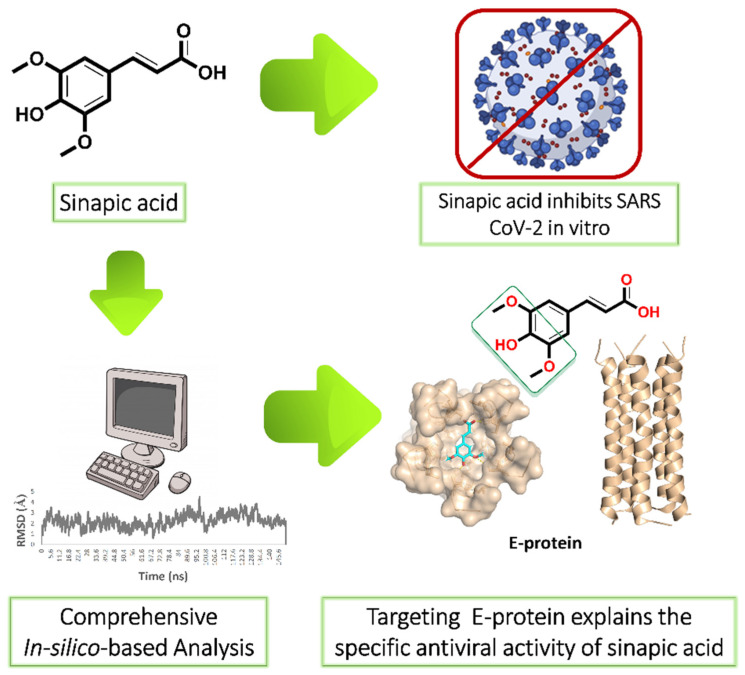
Applied strategy in present study. E-protein: envelope protein.

Our ongoing antiviral screening of different natural compounds indicated that sinapic acid (SA) exhibited a promising selective in vitro antiviral potential. Hence, we tentatively describe the mode of action of sinapic acid (SA) against SARS CoV-2 by applying an in silico approach (Figure 1) as the following: (i) ensemble reverse docking against all currently available SARS CoV-2 proteins to select the promising molecular targets; (ii) validation of the docking results by molecular dynamic simulation (MDS) experiments (150 ns); (iii) performing an extensive literature review on the top-scoring proteins to find out which one has a direct role in the viral in vitro replication; (iv) performing further directed MDS experiments to explore precisely the binding path and mode of SA. We hope that both the experimental and in silico results presented in this study help in providing a potential structural motif for an anti-SARS CoV-2 lead.

## 2. Results

**Figure 2:**
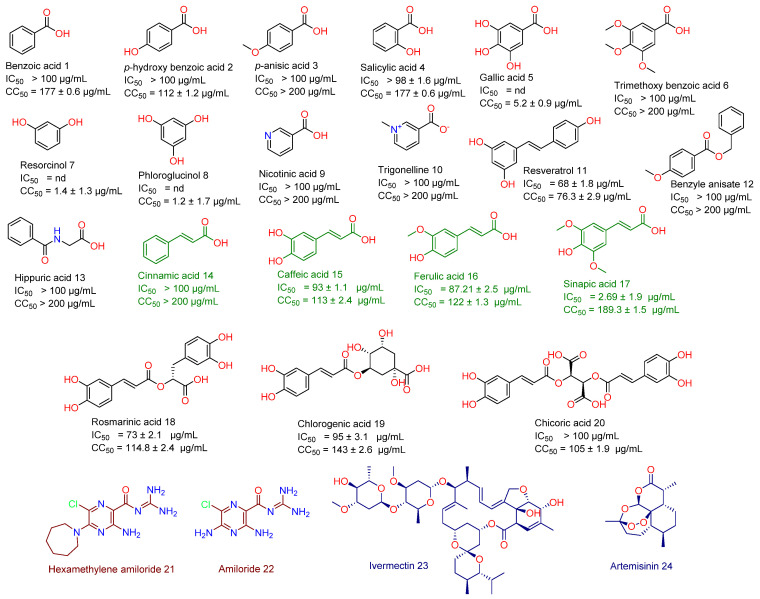
Structures of broccoli-derived compounds (1–20) screened in this study. Green-colored compounds (14–17) are cinnamic acid derivatives that were comprehensively studied in this investigation as a promising anti-SARS CoV scaffold. Brick red-colored compounds (21 and 22) are related synthetic compounds previously studied as SARS CoV inhibitors. Blue-colored compounds (23 and 24) are examples of natural products that have been recently reported as an anti-SARS CoV-2 agent.

**Figure 3:**
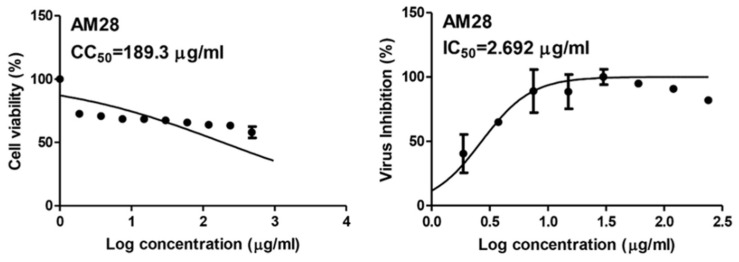
IC_50_ and CC_50_ of sinapic acid (SA).

As a part of our continuous effort to find safe and effective anti-SARS CoV-2, we screened a number of phenolic derivatives derived from broccoli (Figure 2, 20 compounds) for their in vitro viral inhibitory activity. Among the tested phenolics, SA was found to exhibit the most potent SARS CoV-2 inhibition with an half-maximal inhibitory concentration (IC_50_) value of 2.69 µg/mL with significantly low cell toxicity (CC_50_ = 189.3 µg/mL; Figure 2 and Figure 3). Although cinnamic acid (CinA), caffeic acid (CA), and ferulic acid (FA) are structurally related to SA, they were inactive in our in vitro screening (IC_50_ > 50 µg/mL). Such findings indicated that the substitution on the cinnamic acid scaffold’s phenyl moiety could greatly affect the antiviral activity of this class of compounds.

To putatively find out a probable molecular target, we virtually screened SA against all currently available proteins that were reported to be involved in the viral life cycle (https://swissmodel.expasy.org/repository/species/2697049, accessed on 23 December 2020; https://www.genome.jp/kegg-bin/show_pathway?hsa05171+H02398, accessed on 23 December 2020; Table S1). Viral envelope protein (E-protein), ADP ribose phosphatase (ADRP), and cyclin-G associated kinase (GAK) were the only proteins that obtained docking scores with SA <−6 kal/mol (ΔG_vina_ = −8.9, −7.1 and −6.2 kal/mol, respectively). Further MDS validation experiments revealed that SA is unstable inside GAK’s binding site and easily dissociated from it after 12.7 ns, while it was significantly stable inside the E-protein and ADRP binding pockets during the course of 150 ns of MDS (RMSD ~ 1.4 and 2.6 Å, respectively) achieving a binding free energy (ΔG) of −8.4 and −9.3 kcal/mol, respectively.

**Figure 4:**
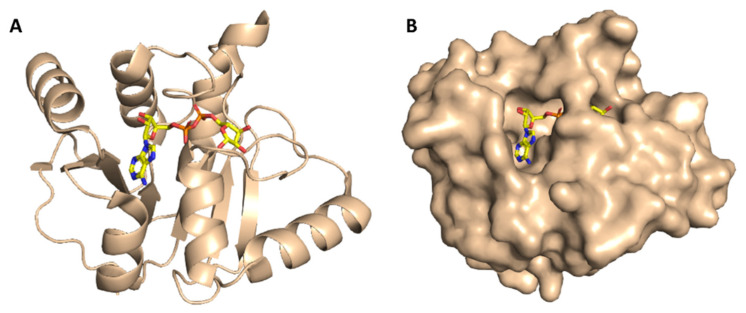
Cartoon and Van der Waals surface representation of ADP ribose phosphatase (ADRP) ((**A**,**B**), respectively) showing the co-crystalized substrate (ADPr) inside its active site.

ADRP (aka macrodomain or MacroD; Figure 4) is expressed as a part of the viral multi-domain non-structural protein-3 (NSP3) and was found to play a crucial role in inhibiting the host innate immunity [19]. However, the deletion or inhibition of this enzyme usually did not inhibit the viral replication in vitro [20].

**Figure 5:**
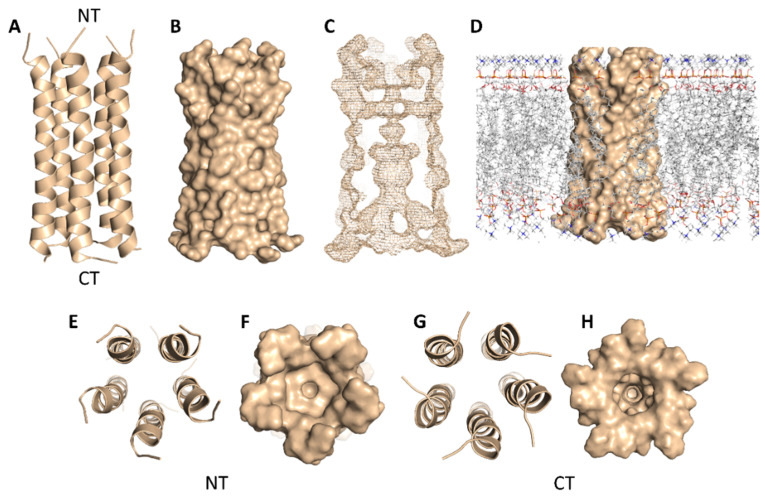
Cartoon and Van der Waals surface representations of the E-protein pentamer ((**A**,**B**), respectively). The geometry of the formed channel across the E-protein pentamer (**C**); Van der Waals surface representation of the E-protein pentamer across the lipid bilayer (**D**); cartoon and Van der Waals surface representations of the E-protein pentamer from the top (**E**,**F**) and bottom (**G**,**H**) views. NT = N terminus and CT = C terminus.

SARS-CoV-2’s E-protein is a 31-residue viroporin that is able to form a cation-selective channel (Figure 5) across the Endoplasmic Reticulum Golgi Intermediate Compartment (ERGIC) and mediates the budding and subsequently the release of the newly formed viruses [21]. Hence, blocking this channel by suitable ligands would result in cation conductance inhibition and reduced viral replication and pathogenicity both in vivo and in vitro [21].

**Figure 6:**
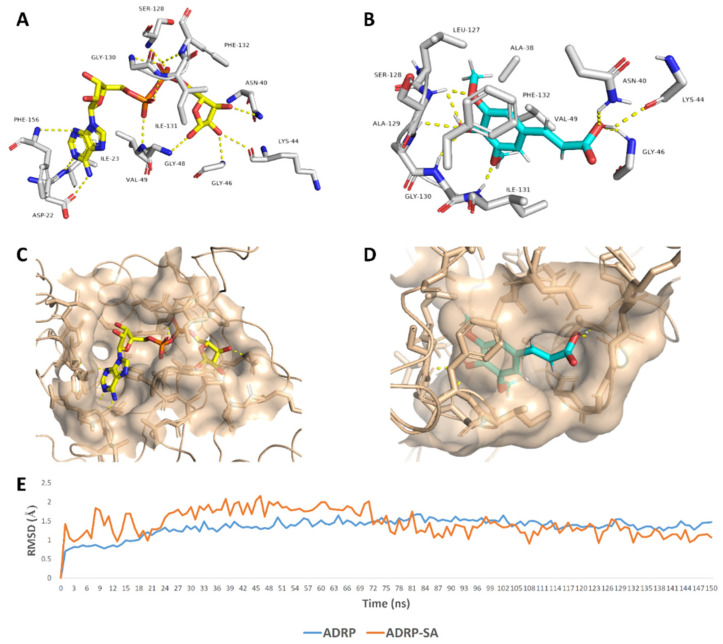
Binding mode of the ADRP’s co-crystalized ligand (ADPr) (**A**,**C**), and SA (**B**,**D**) inside ADRP’s active site. In addition to the RMSDs of ADRP and SA during the course of molecular dynamic simulation (MDS) (**E**).

SA was able to achieve a binding mode comparable with the co-crystalized ligand inside the ADRP’s active site (Figure 6), where it occupied the distal ribose and diphosphate binding sites (R1, P) [19]. SA carboxylate moiety was able to interact with three amino acid residues (AS-40, LYS-44, and GLY-46) via three strong H-bonds (<2.5 Å) similarly to the distal ribose moiety of the enzyme substrate (ADPr) (Figure 6). At the same time, the aromatic part of SA was highly stabilized inside the P-site through a network of H-bonds between the phenolic hydroxy and methoxy groups and the peptidic NH groups of the following residues: SER-128, ALA-129, GLY-130, and ILE-131. Besides, both methyl groups that attach to the benzene ring via two etheric bonds were involved in hydrophobic interactions with ALA-38, VAL-49, and LEU-127’s side chains, and the benzene ring was further stabilized via the hydrophobic interaction with ILE-131’s side chain and via π–π staking with the PHE-132’s benzene ring. All these molecular interactions inside the cervices of the ADRP active site remained intact throughout the course of MDS (RMSD~1.4 Å; 150 ns) making SA a remarkably stable potential inhibitor (Figure 6).

**Figure 7:**
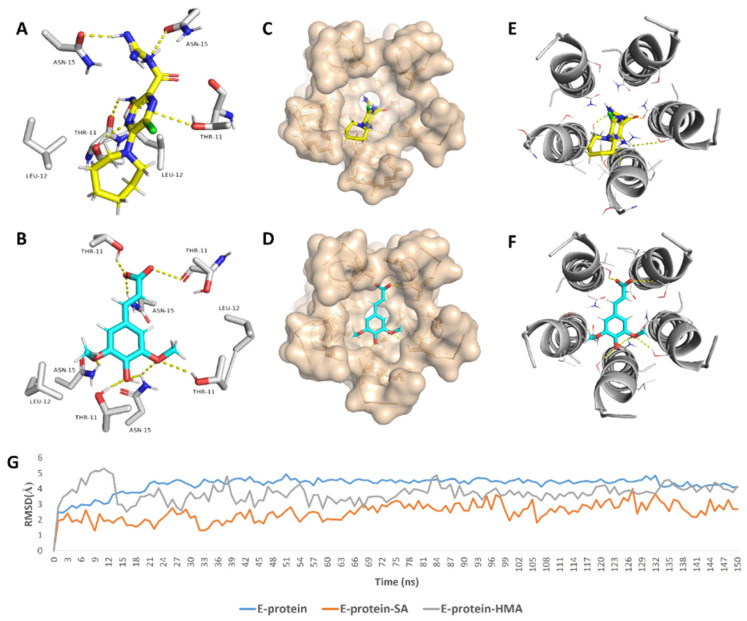
Binding mode of the previously reported inhibitor (hexamethylene amiloride—HMA) and SA inside the E-protein’s channel opening (N-terminus) ((**A**,**B**), respectively) indicating the involved helices (**C**–**F**). In addition to the RMSDs of E-protein, SA and HMA during the course of MDS (**G**).

**Figure 8:**
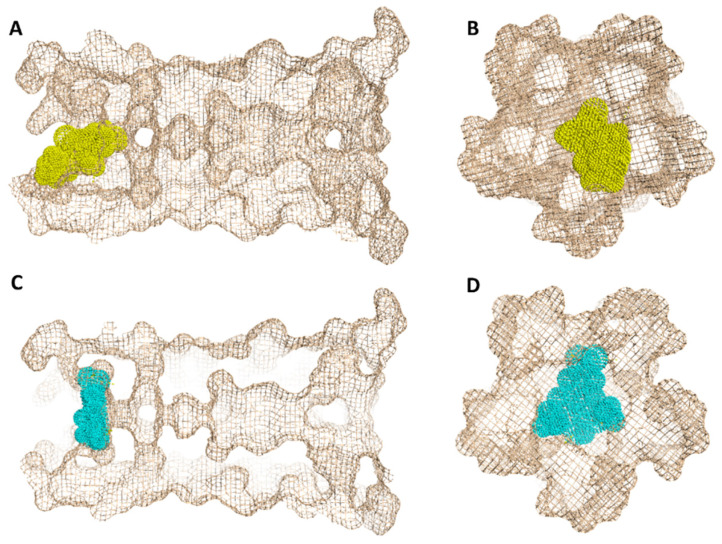
Geometry of the E-protein’s channel showing the orientation of both the previously reported inhibitor (HMA) (**A**,**B**) and SA (**C**,**D**) and how they block the channel’s opening.

In regard to E-protein, it normally assembles into a trans-membrane homopentamer cation-selective channel (Figure 5). The N-terminal pore of this channel has been previously reported as a binding site for a number of ligands, e.g., hexamethylene amiloride (HMA) [21,22,23]. This type of binding site is highly dynamic due to being an ensemble of multiple subunits, and the high RMSD and RMSF values of the whole channel including the N-terminal pore are good evidence of such flexibility (Figure S1). SA achieved an interesting docking pose (Figure 7) with a high score inside this binding site that was even better than that of the previously reported inhibitor HMA, where it was able to bridge the five helices (Figure 6). The unique scaffold of SA makes it able to be perfectly fitted inside the pore opening, producing complete occlusion (Figure 7 and Figure 8). Similar to HMA, SA was able to form two H-bonds with THR-11 of two adjacent helices through one of the meta-methoxy groups and the phenolic hydroxy group which was also H-bonded to ASN-15 from one helix, while the second methoxy group was H-bonded ASN-15 from the adjacent helix (Figure 7). In addition, the methyl moiety of the two methoxy groups interacted with LEU-12 from separate helices via hydrophobic interactions. Hence, the aromatic part of SA was able to bridge three adjacent helices. The carboxylate moiety formed H-bond with ASN-15 with one of the remaining helices similarly to HMA. Moreover, it also H-bonded to THR-11 of these remaining helices bridging them together. This unique orientation and binding mode of SA inside the N-terminal’s pore remained stable throughout 150 ns of MDS (RMSD~2.6 Å) regardless of the obvious instability of this binding site (Figure 7G), while that of HMA was dynamic and interchangeable between helices.

**Figure 9:**
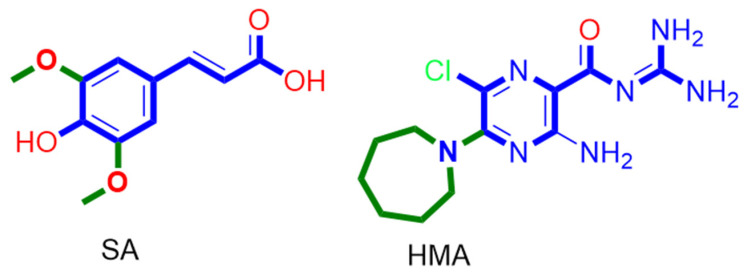
Structures of SA and HMA indicate the importance of the presence of a bulky hydrophobic moiety for their antiviral activity.

The structurally related derivatives CinA, CA and FA have binding modes convergent with that of SA. However, the lack of a third substituent on the benzene ring made them attach only to four helices (for CA and FA), and not orient deeply inside the pore-like HMA; they were easily dissociated from this channel opening (Figure S4). Such dissociation was not observed in SA thanks to the bulky 3,5-dimethoxy groups that played a crucial role in this regard and kept the aromatic part of the molecule stabilized inside the binding site. Similarly, removing the bulky hexamethylene group was previously reported to be responsible for the loss of HMA’s antiviral activity [22,23]. However, upon MDS, we found that the amiloride part was unstable and completely left the binding site at 83 ns (Figure S2) just like CinA, FA and CA, indicating that such hydrophobic moieties stabilize the whole ligand inside the binding site. Consequently, these structural and dynamic information might explain the antiviral activity of SA and the inactivity of its closely related counterparts CinA, CA and FA (Figure 9).

**Figure 10:**
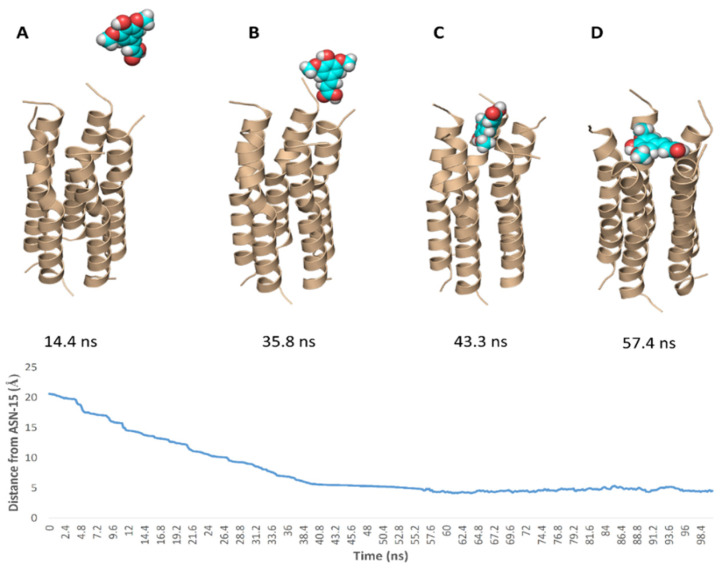
Simulation of the binding path of SA (**A**–**D**), where it reached a binding mode and orientation similar to that of the docking pose at 43.2 ns.

To simulate the binding of SA with this N-terminal pore, we conducted a number of MDS experiments by directing one molecule of SA toward this binding site and applying a force at each trial. Interestingly, we were able to produce a binding event (Figure 10) which revealed the SA path and binding mode with the pore opening. Firstly, the carboxylate moiety interacted with GLY-8 and THR-9 of one helix via H-bonds, and gradually the whole molecule turned around. Then, the tri-substituted benzene moiety faced the pore opening and slowly landed on it and bridged three helices. Subsequently, the molecule front, i.e., the carboxylate moiety also landed bridging the remaining helices and finally, the whole molecule achieved a binding mode similar to that of the docking pose.

## 3. Discussion

Despite the development of several vaccines in late 2020 and early 2021 against SARS-CoV-2 as a prophylactic measure, finding a proper antiviral agent is still of significant priority, particularly in countries of weak economies. 

Besides the investigation of synthetic small molecules, natural products are also a crucial pipeline for promising safe, effective, and cheap antiviral agents. The rapid and continuous characterization of SARS-CoV-2 proteins plays a fundamental role in the development of potential therapeutics. Now, most of the viral components and protein–protein interactions are well-characterized, and hence, structural-based virtual screening trials can be conducted. ADRP and E-protein are among the recently characterized SARS-CoV-2 molecular targets. ADRP inhibitors have been previously proven to activate the host immune response against SARS-CoV-2 infection in vivo. However, they were not able to inhibit the viral replication in vitro, indicating that the central role of this protein is to facilitate the viral escape from the host immunity, but it has no direct impact on its replication [20]. On the other hand, the viral E-protein was proven to be a crucial viral protein not only in coronaviruses but also in other envelope-based viruses [21,22,23]. This short helical protein is able to aggregate in pentamers, forming a cation-selective channel across lipid bilayers and inside the viral envelope. Despite the fact that the exact role of this protein in the viral life cycle is still elusive, blocking the conductance of this channel by small molecules (e.g., HMA and amantadine) was associated with the inhibition of the viral replication in vitro [21,22,23].

Phenolic compounds are considered one of the most abundant secondary metabolites in the plant kingdom and have previously shown therapeutic potential against many pathological conditions, including viral infections [2,18]. Herein, we screened a number of phenolic natural products for their in vitro inhibitory activity against SARS-CoV-2, where SA was found to achieve a remarkable antiviral effect (IC_50_ 2.67 µg/mL) with minimal cell toxicity (CC_50_ 189.3 µg/mL). To explore the probable target of SA, we utilized all the currently available SARS CoV-2 protein structures in an inverse ensemble docking protocol. Both ADRP and E-protein were the best scoring hits for SA but also for the inactive related derivatives CinA, CA and FA. Being the best scoring protein that its inhibition has a direct link to the viral inhibition in vitro as discussed earlier, we subjected SA, CinA, CA, and FA complexes with E-protein to a series of MDS experiments to explain the mode of interaction of the three congeners. The presence of 4-hydroxy alongside 3,5-dimethoxy substituents in the benzene ring of SA appeared to be an essential structural motif for keeping the whole molecule intact at the highly flexible channel binding site that in the absence of bulky substituents at one end of the inhibitor molecule was able to juggle itself significantly until the complete dissociation. Moreover, we simulated the probable binding path of SA and how it landed and fitted inside the channel binding pore. SA is among the chief constituents in broccoli florets and sprouts (3.43 to 140.53 mg/100 g dry weight) [24] and has shown many pharmacological activity and health benefits [25,26,27,28].

In conclusion, we suggest SA as a promising simple and easily accessible lead compound for further in vivo and clinical investigations and future structural optimization to provide more potent broad-spectrum antiviral therapeutics.

## 4. Materials and Methods

### 4.1. Isolation of Compounds 

All plant-derived phenolic compounds (20 compounds, Figure 2) used in this study were isolated from broccoli (obtained from a local market). Briefly, 2 kg of fresh broccoli florets were extracted four times by 95% ethanol. Subsequently, the produced extract was concentrated under vacuum to give 50 g of crude extract and then it was chromatographed on silica gel while using CH_2_Cl_2_/MeOH (50:50) to afford 6 fractions. Further chromatographic purification of fractions 3–6 on silica gel column used a gradient solvent from CH_2_Cl_2_ 100% to MeOH 100%. The produced sub-fractions were finally purified on Agilent^®^ 1260 Infinity semi-preparative HPLC using acetonitrile in water (60–100%) for 30 min followed by 100% acetonitrile for the next 20 min at a 7 mL/min flow rate to afford 20 different phenolic compounds (1–20, Figure 2) that were characterized by spectroscopic means (e.g., ^1^H-NMR, Figures S3–S5) and comparison with authentic standards. CinA, FA, CA, and SA were obtained in good quantities (40.2, 22.5, 51.6, 112 mg, respectively). All solvents, reagents and other chemicals were obtained from Sigma Aldrich (Paisley, UK) and Santacruz Biotech (California, USA). SA and its derivatives were also purchased from Sigma Aldrich and/or Santacruz Biotech to ensure the maximum purity (>98% purity) during the in vitro testing.

### 4.2. Data Preparation

Regarding the SARS CoV-2 proteins, all currently available viral and non-viral proteins relevant to the COVID-19 were retrieved from the Swiss-Model repository (https://swissmodel.expasy.org/repository/species/2697049, accessed on 23 December 2020) and String database (https://string-db.org/cgi/covid.pl, accessed on 23 December 2020) [9,29]. All the PDB codes of the proteins used in this study along with their abbreviations and functions are listed in Table S1.

### 4.3. Ensemble Docking 

We used AutoDock Vina software in all docking experiments [30]. SA along with its related derivatives cinnamic acid (CinA), caffeic acid (CA) and ferulic acid (FA) were docked against all the collected proteins (their PDB codes are listed in Table S1). The binding site of each protein was determined according to its co-crystalized ligand. Homology models along with other proteins without co-crystallized ligands were subjected to a blind docking protocol in which the software predicted all possible binding sites. To account for these proteins’ flexibility, we used their MDS-derived conformers sampled every 10 ns for docking experiments (i.e., ensemble docking) [4]. Subsequently, we ranked the resulting top hits according to their calculated binding energies. Docking poses were analyzed and visualized by Pymol software [30].

### 4.4. Molecular Dynamic Simulation

MDS were performed by Desmond v. 2.2 [31,32] the MDS machine of Maestro software [33] using the OPLS force field. Protein systems were built via the System Builder option, where it was embedded in an orthorhombic box of TIP3P waters together with 0.15 M Na^+^ and Cl^−^ ions with 20 Å solvent buffer. Afterwards, the prepared systems were energy minimized and equilibrated for 10 ns.

Desmond software automatically parameterizes inputted ligands during the system building step according to the OPLS force field. For simulations performed by NAMD [34], the parameters and topologies of the compounds were calculated either using the Charmm27 force field by the online software Ligand Reader and Modeler (http://www.charmm-gui.org/?doc=input/ligandrm, accessed on 23 December 2020) [35] or by the VMD plugin Force Field Toolkit (ffTK) [36]. Afterwards, the generated parameters and topology files were loaded to VMD so that it can readily read the protein–ligand complexes without errors and then conduct the simulation step.

Binding free energy calculations (Δ*G*) were performed using the free energy perturbation (FEP) method. We first prepared the input files and script NAMD by the online-based software CHARMM-GUI Free Energy Calculator (http://www.charmm-gui.org/input/fec, accessed on 23 December 2020) [35]. Afterwards, these inputs were loaded to NAMD for simulations, where the equilibration was performed in the NPT ensemble at 300 K and 1 atm (1.01325 bar) with Langevin piston pressure (for “Complex” and “Ligand”) in the presence of the TIP3P water model. 10 ns FEP simulations were performed for each compound, and the last 5 ns of the free energy values was measured for the final free energy values [35,36]. For further confirmation of the initial docking and MDS experiments, we generated a binding event simulation by placing the ligand close to the E-protein’s binding site (21 Å away from ASN-15) and applying force (10 kcal/mol · A^2^) toward its center to make SA move toward the binding site with a velocity of 0.31 Å/ns. Finally, generated trajectories were visualized and analyzed by VMD software [36,37].

### 4.5. In Vitro Antiviral Assay

#### 4.5.1. Virus and Cells

Dulbecco’s Modified Eagle’s medium (DMEM) with 10% fetal bovine serum (FBS) (Invitrogen) and 2% penicillin/streptomycin mixture was used to maintain Vero-E6 cells at 37 °C, 5% CO_2_. Virus stock was prepared by distributing cells into tissue culture flasks 24 h before infection with hCoV-19/Egypt/NRC-3/2020 isolate at a multiplicity of infection (MOI) of 0.1 in an infection medium (DMEM) containing 4% FBS, 1%, 1% L-1-tosylamido-2-phenylethyl chloromethyl ketone (TPCK)-treated trypsin and penicillin/streptomycin). After two hours, the infection medium containing virus inoculum was exchanged with freshly infected medium and incubated for three days. Cell supernatant was thereafter collected and centrifuged for 5 min at 2500 rpm from purification. Afterwards, the supernatant was transferred to a fresh 50 mL falcon tube, aliquoted, and then titrated using plaque infectivity assay.

#### 4.5.2. MTT Cytotoxicity Assay

To determine the half-maximal inhibitory concentration (IC_50_) to be used for the initial assessment of the compounds for their antiviral screening, we prepared stock solutions of the extracts and test compounds in 10% DMSO with ddH_2_O and further diluted to the working solutions with DMEM. The cytotoxic effect of the test compounds was evaluated in Vero-E6 cells by using the previously reported 3-(4,5-dimethylthiazol-2-yl)-2,5-diphenyltetrazolium bromide (MTT) method [38] with minor modifications. Briefly, the cells were placed in 96-well plates (100 µL/well at a density of 3 × 10^5^ cells/mL) and then incubated for 24 h at 37 °C in 5% CO_2_. After 24 h, cells were treated with different tested compounds concentrations in triplicates. After another 24 h, the supernatant was discarded, and cell monolayers were washed with sterile 1 x PBS 3 times. MTT solution (20 µL of 5 mg/mL stock solution) was added to each well and then incubated at 37 °C for 4 h. The produced formazan crystals were dissolved with 200 µL of acidified isopropanol (0.04 M HCl in absolute isopropanol = 0.073 mL HCL in 50 mL isopropanol). After that, we measured the absorbance of formazan solutions at λ_max_ 540 nm using a multi-well plate reader. The percentage of cytotoxicity compared to the untreated cells was determined with the following equation:

(1) $$\%\;\mathrm{cytotoxicity}=\frac{\left(\mathrm{absorbance}\;\mathrm{of}\;\mathrm{cells}\;\mathrm{without}\;\mathrm{treatment}-\mathrm{absorbance}\;\mathrm{of}\;\mathrm{cells}\;\mathrm{with}\;\mathrm{treatment}\right)\;\times 100}{\mathrm{absorbance}\;\mathrm{of}\;\mathrm{cells}\;\mathrm{without}\;\mathrm{treatment}\;}$$

The produced plot of % cytotoxicity versus sample concentrations was then used to calculate the IC_50_s.

#### 4.5.3. Viral Inhibitory Concentration 50 (IC_50_) Determination

Approximately 2.4 × 10^4^ Vero-E6 cells were placed in each well of a 96-well tissue culture plates and incubated overnight in a humidified incubator at 37 °C under 5% CO_2_ condition. Then, cell monolayers were washed once with 1× PBS and subjected to virus adsorption for 1 h at room temperature. Afterwards, the cell monolayers were overlaid with 50 μL of DMEM containing different tested compounds concentrations. Following incubation for 72 h, the cells were fixed with 100 μL of 4% paraformaldehyde for 20 min and then stained with 0.1% crystal violet for 15 min. Subsequently, the crystal violet dye was dissolved with 100 μL of methanol per well, and the optical density of the color measured at 570 nm using Anthos Zenyth 200 rt plate reader (Anthos Labtec Instruments, Heerhugowaard, The Netherlands). The IC_50_ of the compound is the concentration that reduces the virus-induced cytopathic effect (CPE) by 50%, relative to the virus control.

## Data Availability

Not applicable.

## Supplementary Materials

The following are available online at https://www.mdpi.com/article/10.3390/antibiotics10040420/s1, Figure S1: Broccoli-derived phenolic compounds screened in this study; Figure S2: RMSDs of SA inside the binding sites of the GAK during the course of 60 ns MDS; Figure S3: RMSD and RMSF of E-protein; Figure S4: RMSDs of cinnamic, ferulic, and caffeic acids along with ameloride inside the binding sites of E-protein during the course of 150 ns MDS; Figure S5: 1H-NMR spectrum of sinapic acid acid in (DMSO-d6); Table S1: All SARS CoV-2 used in this study.
