# Supplementary material for: Sinapic Acid Suppresses SARS CoV-2 Replication by Targeting Its Envelope Protein
Source: Antibiotics (Basel). 2021 Apr 11;10(4):420. doi: 10.3390/antibiotics10040420 (PMC8069661; doi:10.3390/antibiotics10040420)
Supplement: Supplementary file 1 [file antibiotics-10-00420-s001.pdf]

antibiotics

Supplementary Material

Sinapic acid Suppresses SARS CoV-2 Replication by Targeting its Envelope Protein

Raha Orfali [1], Mostafa E. Rateb [2], Hossam M Hassan [3,4], Mona Alonazi [5], Mokhtar R. Gomaa [6], Noura Mahrous [6], Mohamed GabAllah [6], Ahmed Kandeil [6], Shagufta Perveen [1], Usama Ramadan Abdelmohsen [7,8,]* and Ahmed M. Sayed [3,]*

[1] Department of Pharmacognosy, College of Pharmacy, King Saud University. P. O. Box 22452, Riyadh 11495, Kingdom of Saudi Arabia, rorfali@ksu.edu.sa, shakhan@ksu.edu.sa.
[2] School of Computing, Engineering & Physical Sciences, University of the West of Scotland, Paisley PA1 2BE, United Kingdom. Mostafa.Rateb@uws.ac.uk.
[3] Department of Pharmacognosy, Faculty of Pharmacy, Nahda University, Beni-Suef 62513, Egypt, hossam.mokhtar@nub.edu.eg.
[4] Department of Pharmacognosy, Faculty of Pharmacy, Beni-Suef University, Beni-Suef 62513, Egypt.
[5] Department of Biochemistry, Faculty of Science, King Saud University. P. O. Box 12372, Riyadh 11495, Kingdom of Saudi Arabia. moalonazi@Ksu.edu.sa.
[6] Center of Scientific Excellence for Influenza Virus, Environmental Research Division, National Research Centre, Giza 12622, Egypt. Emails: noura.mahrous1995@gmail.com; gaballah09@gmail.com; Mokhtar.Rizk@human-link.org; Kandeil_a@hotmail.com.
[7] Department of Pharmacognosy, Faculty of Pharmacy, Minia University, Minia 61519, Egypt, usama.ramadan@mu.edu.eg.
[8] Department of Pharmacognosy, Faculty of Pharmacy, Deraya University, 7 Universities Zone, New Minia 61111, Egypt, Ahmed.mohamed.sayed@nub.edu.eg.

Protein	Name	Function	PDB Code
**Viral Proteins (Nonstructural proteins)**			
nsp1	-	Host translation inhibitor	7K3N
nsp2	-	Play a role in the modulation of host cell survival signaling pathway by interacting with host PHB and PHB2	Deposited model in Swiss-Model
nsp3	ADP ribose phosphatase	Activate replicase polyprotein	6W02
nsp4	-	Participates in the assembly of virally-induced cytoplasmic double-membrane vesicles necessary for viral replication	Deposited model in Swiss-Model
nsp5 (3CL-PRO)	Main protease	Cleaves the C-terminus of replicase polyprotein at 11 sites	6lu7
nsp6	-	Plays a role in the initial induction of autophagosomes	Deposited model in Swiss-Model
nsp7	-	Plays a role in viral RNA synthesis	6M5I
nsp8	-	Plays a role in viral RNA synthesis	6M5I
nsp9	-	May participate in viral replication by acting as a ssRNA-binding protein	6WC1
nsp10	-	plays an essential role in viral mRNAs cap methylation	6ZPE
nsp11 (PL-PRO)	Papain-like protease	Participate in the activation of the replicase polyprotein	6WX4
nsp12 (Pol; RdRP)	RNA-directed RNA polymerase	Responsible for replication and transcription of the viral RNA genome	6M71
nsp13 (Hel)	Helicase	Displaying RNA and DNA duplex-unwinding activities with 5' to 3' polarity. Activity of helicase is dependent on magnesium	5RL6
nsp14 (ExoN)	Proofreading exoribonuclease	An exoribonuclease activity acting on both ssRNA and dsRNA in a 3' to 5' direction	Deposited model in Swiss-Model

Nsp15 (NendoU)	Uridylate-specific endoribonuclease	Mn(2+)-dependent, uridylate-specific enzyme, which leaves 2'-3'-cyclic phosphates 5' to the cleaved bond.	5S6X
nsp16	2'-*O*-methyl-transferase	Methyltransferase that mediates mRNA cap 2'-O-ribose methylation to the 5'-cap structure of viral mRNAs	6W4H
**Structural proteins**			
S glycoprotein	Spike glycoprotein	Binding to human ACE2 receptor and internalization of the virus into the endosomes of the host cell induces conformational changes in the Spike glycoprotein	6lzg and 6zb5
E-protein	Envelope small membrane protein	Plays a central role in virus morphogenesis and assembly	7k3g
M-protein	Membrane protein	Component of the viral envelope that plays a central role in virus morphogenesis and assembly via its interactions with other viral proteins	Deposited model in Swiss-Model
N-protein	Nucleoprotein	Packages the positive strand viral genome RNA into a helical ribonucleocapsid (RNP) and plays a fundamental role during virion assembly through its interactions with the viral genome and membrane protein M	6M3M
**Accessory Proteins**			
ORF3a	ORF3a protein	Forms homotetrameric potassium sensitive ion channels (viroporin) and may modulate virus release	6XDC
ORF6	ORF6 protein	Disrupts cell nuclear import complex formation by tethering karyopherin alpha 2 and karyopherin beta 1 to the membrane	Deposited model in Swiss-Model
ORF7a	ORF7a protein	Plays a role as antagonist of host tetherin (BST2), disrupting its antiviral effect	6w37
ORF7b	ORF7b protein	-	-
ORF8	ORF8 protein	May play a role in host-virus interaction	7JTL
ORF9b	ORF9b protein	Plays a role in the inhibition of host innate immune response by targeting the mitochondrial-associated adapter MAVS	6Z4U
**Human-derived Proteins**			
ACE2	Angitensin converting enzyme 2	Responsible for attachment of SARS CoV	6LZG
Furin	Furin	Activation of S-protein	6EQX
TMPRSS2	Transmembrane protease, serine 2	Activation of S-protein	Deposited model in Swiss-Model
CatL	Cathepsin L	Activation of S-protein	2YJC
NPR1	Neuropilin 1	Facilitates viral entry	3i97
PHB1	Prohibitin subunit1	In the mitochondria, together with PHB2, forms large ring complexes (prohibitin complexes) in the inner mitochondrial membrane (IMM) and functions as chaperone protein that stabilizes mitochondrial respiratory enzymes and maintains mitochondrial integrity	Deposited model in Swiss-Model
PHB2	Prohibitin subunit2	In the mitochondria, together with PHB2, forms large ring complexes (prohibitin complexes) in the inner mitochondrial membrane (IMM) and functions as chaperone protein that stabilizes mitochondrial respiratory enzymes and maintains mitochondrial integrity	Deposited model in Swiss-Model
AAK1	Adaptor Protein 2 Associated Kinase	Participate in viral endocytosis	4WSQ
GAK	Cyclin-G associated kinase	Participate in viral endocytosis	4y8d

Table S1. All SARS CoV-2 related proteins used in this study.

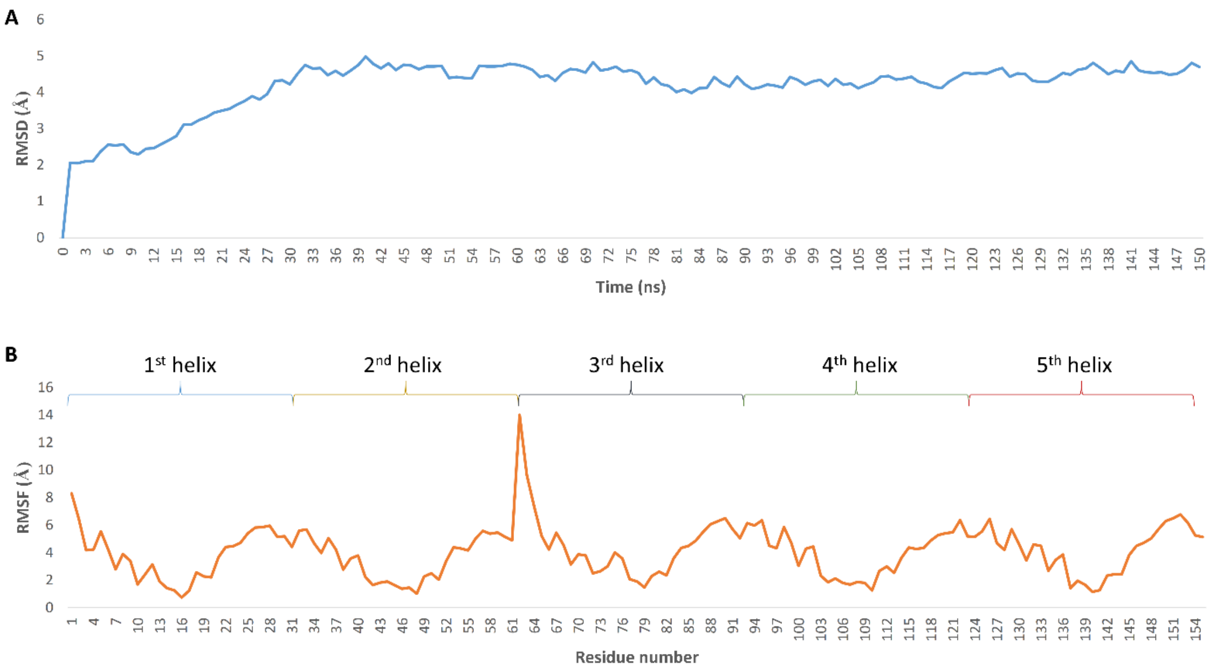

Figure S1. RMSD and RMSF of E-protein.

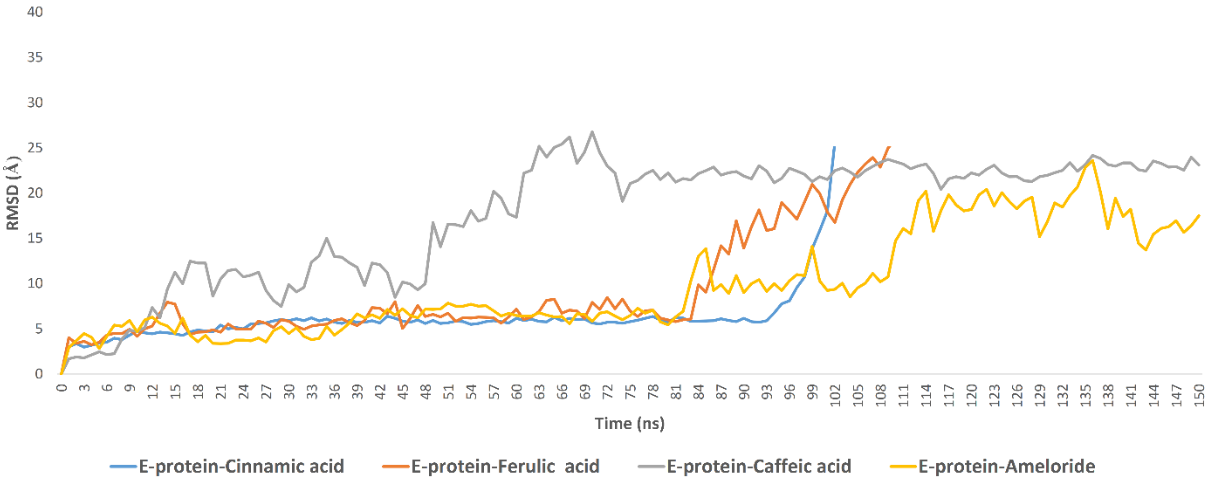

Figure S2. RMSDs of cinnamic, ferulic, and caffeic acids along with ameloride inside the binding sites of E-protein during the course of 150 ns MDS.

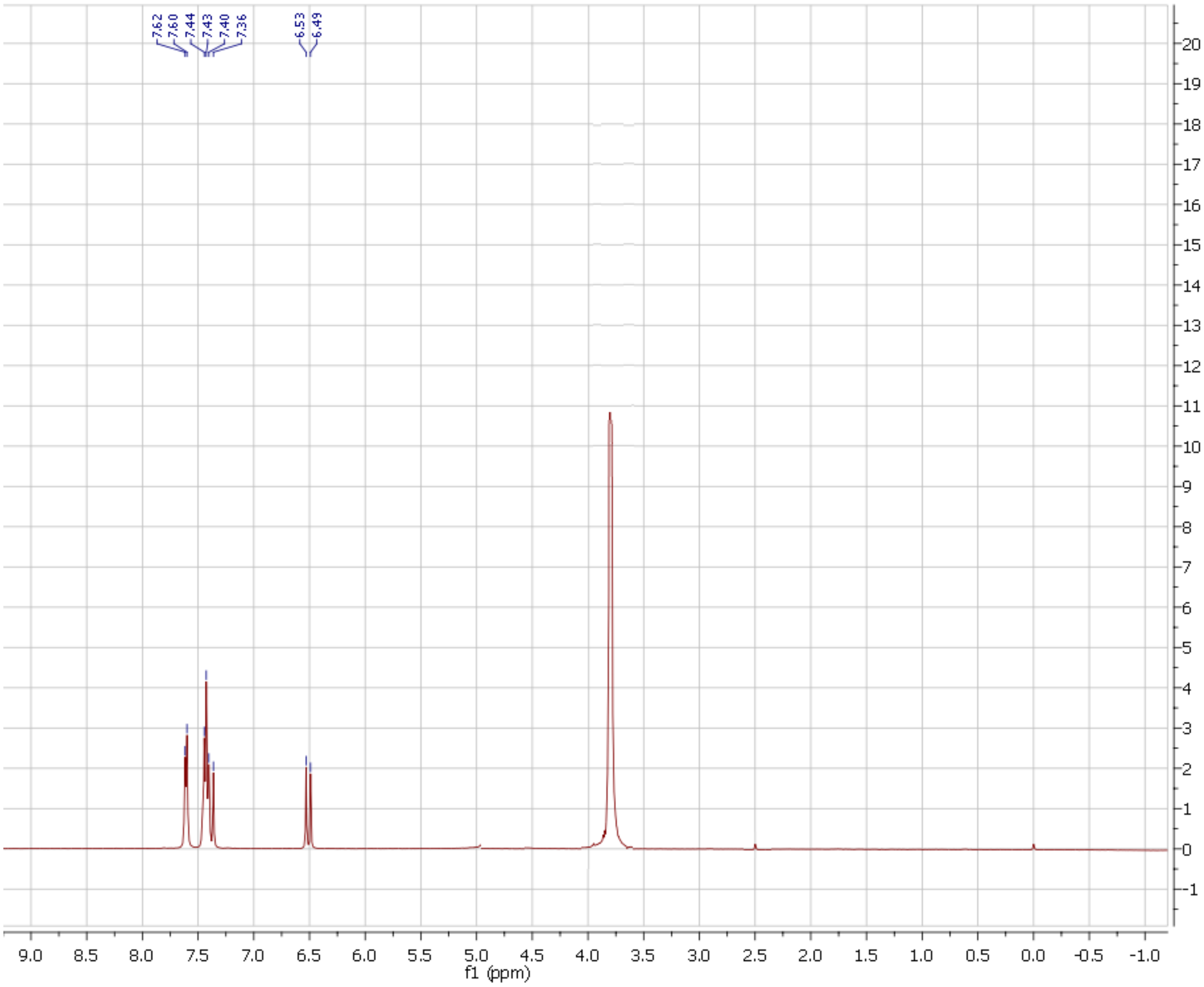

Figure S3. ^{1}H-NMR spectrum of cinnamic acid in (DMSO-d_6).

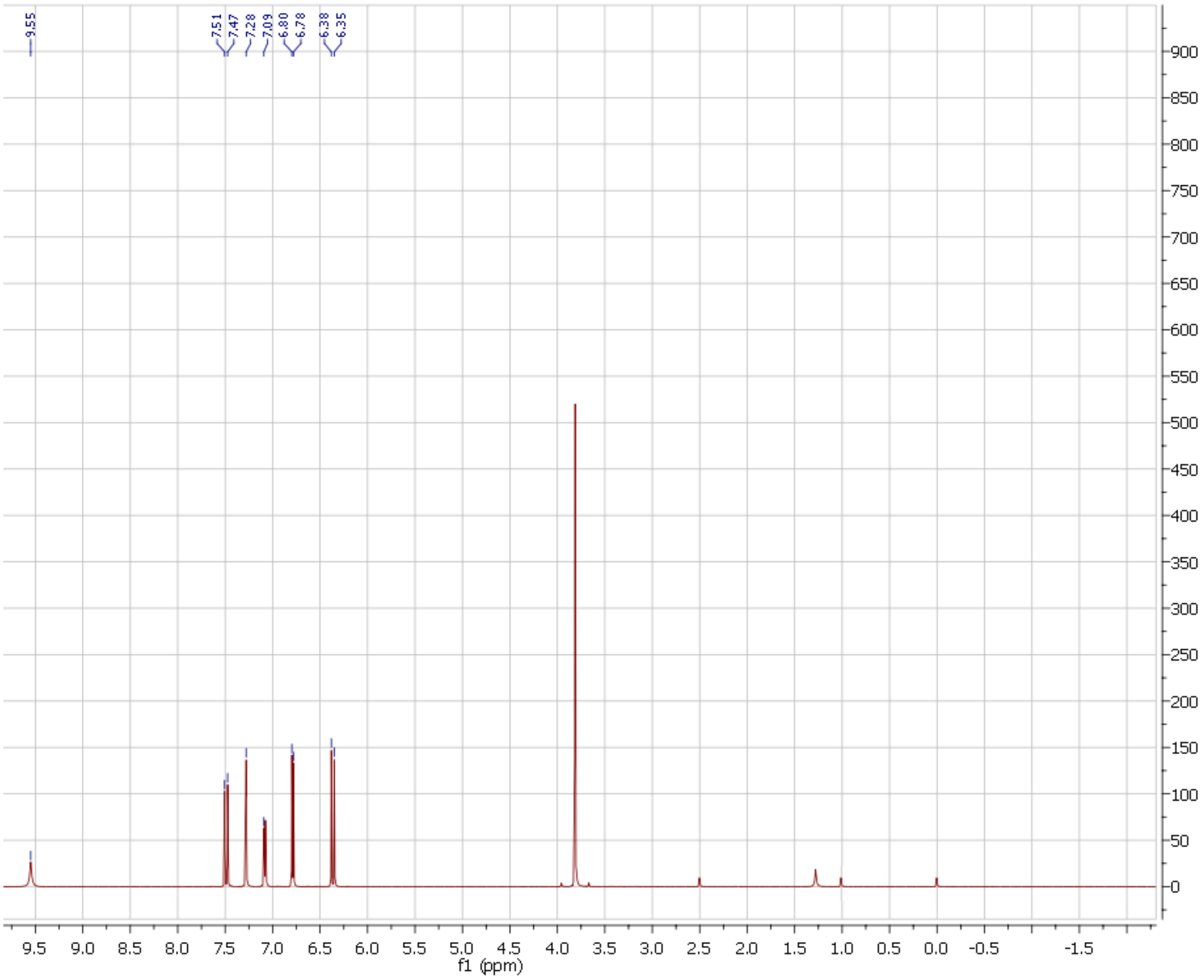

Figure S4. ^{1}H-NMR spectrum of ferulic acid in (DMSO-d_6).

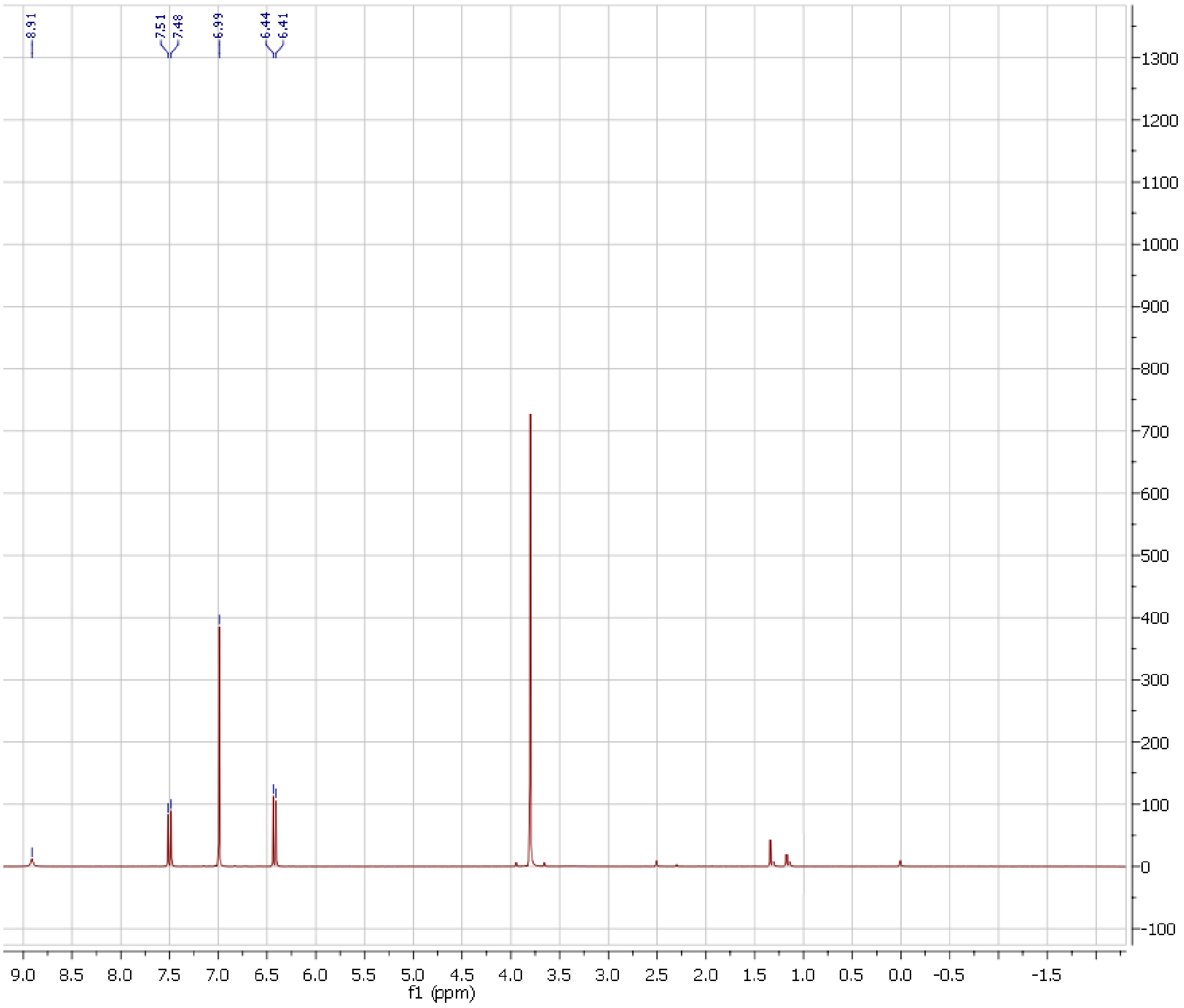

Figure S5. ^{1}H-NMR spectrum of sinapic acid acid in (DMSO-d_6).
